# Treatment of Dental Pulp Preparatory to Plugging

**Published:** 1850-04

**Authors:** J. D. White

**Affiliations:** Dentist


[From the Dental News Letter.]

### TREATMENT OF DENTAL PULP PREPARATORY TO PLUGGING.

BY J. D. WHITE, M. D., DENTIST.

*Of false Tooth-ache.*—Acute sensibility, by striking against the crown of the tooth with an instrument, especially upon the face, and in cases of teeth that have more than one root, striking upon the cusps opposite, and in the direction of the roots separately, will lead to a direct diagnosis, which is inflamed, or which is acutely affected. This precaution should never be omitted, as it is often that only one root of two or more is diseased. A seeming elongation of the tooth from the socket, and more yielding motion than in healthy teeth in the same mouth; these latter symptoms may be more or less marked, proportionately to the *hyperemia* and thickening of the periosteal membranes, and an abscence of pain by passing an instrument into the pulp cavity, together with an insensibility to pain by applying cold to the parts.

*Of Sympathetic Tooth-ache.*—This may happen to teeth which are wholly sound; but often they are found to have undergone some morbid change, such as recession of the gum from the neck of the tooth, or irritation of the external membranes, excited by salivary calculi, slight decay of the dentine, erosion, or defective in some way or other. On account of the fact, that pain is so frequently experienced in very sound teeth, and in remote parts, such as in the temples, top of the head, ears, and even the shoulders, it would be unpardonable in an operator to extract an aching tooth, until he had formed his diagnosis from the most positive signs, that the pulp was really exposed, or it was the actual seat of the disease. Upon this point, we have occasion daily to exercise the greatest precaution. I will be pardoned for digression, to relate, at this point, a very remarkable case. Master T., of Chester county, aged fourteen years, was brought to me two years ago, from school, suffering very much at times, and especially at night, with pain in the temples, and in both of the superior front incisors, which were partially decayed, but not more than half way from the surface of the enamel to the pulp cavities. I was requested to destroy the nerves and plug them, or extract; anything to get rid of the pain. Upon examination of the mouth generally, I discovered that the nerves were exposed in both of the first inferior molars. I directed their extraction, which was acceeded to, although they had never been the site of pain, nor had they been suspicioned as being any way connected with the patient's suffering. The front teeth were plugged without any trouble, and there has not been any of the former symptoms experienced since.

*Treatment of the Dental Pulp.*—The treatment of the exposed pulp has given rise to great difference of sentiment among well educated dentists; but mainly about the *means* which should be employed for that purpose, agreeing, pretty generally, that it is bad practice to destroy it entirely. But as well might we expect to procure a healthy function of the *rete-mucosum*, when denuded of the *epidermis*, by substituting one of our own invention, as to procure a healthy function of the pulp, when deprived of its natural protection—the bone.—The various modes of treatment, which have for their object the preservation of the pulp, must be of that order. When the pulp becomes exposed by decay, or any other cause, the delicate vessels which ramify upon its surface, are soon ruptured, as well as those which passed into the bone which have been destroyed; they pour out blood and serum, which must have exit through an external opening, or inflammation supervenes, and in a short time establishes a suppurating surface. Any attempt to remove this pathological condition permanently, by medical or mechanical agents, must of necessity prove ineffectual. Notwithstanding, this seems to be the language of reason and experience, it is the object sought to be obtained by most practitioners in dental surgery. I would consider this to be an invariable truth, that *so long as the artery continues to convey blood to the pulp, so long will there exist the necessity for an external opening, effusive, or suppurating surface*, unless the inflammation becomes so violent as to produce a slough of the whole pulp.*

*By Astringents and Capping.*—This is a mode of treatment much extolled by some dentists. Dr. Fitch remarks, "I think the best practice will be, and is, to unite both, as I am in the habit of doing, which is, use the astringents for some time, and then cover the nerve with a cap of lead or gold plate, and complete the filling of the cavity with gold. If this practice be adopted by the dentist, he will often save the tooth."—Yet he frankly admits, that, "in many cases it entirely fails." Now, if the cap could not save the tooth before the astringents were applied to the pulp, how could it do so afterwards? The therapeutist teaches, that the effect of an agent that does not destroy the vitality of the part to which it is applied, is of very short duration. Astringents do not in these cases destroy the vitality of the pulp; then, of course, it may return in a very short time to the same condition in which it was found, before the astringents were used, (nor would the cap be necessary to protect it from pressure,) and give rise to all those dreaded evils which would have followed the application of the cap in the first instance. Professor Harris, of Baltimore, with his usual candor, in speaking of the above method of treatment, says, "It is not recommended as infallible; and

* I would refer the reader for a more full discussion of the physiological and pathological considerations of the teeth, to an address delivered by the writer, and published in "Stockton's Dental Intelligencer," vol. 2, No. 9 July 1st, 1846.

while I declare, that it has been more successful than any other that I have tried, candor compels me to add, that it has failed in more instances than it has succeeded."

This is about the success with which the writer has met, in adopting the above plan of treatment, *or any other method which has for its object the preservation of the vitality of the pulp.* I think I hazard nothing in asserting that as great a number may be saved without any preparatory treatment whatever, if the pulp be not actually pressed upon by the stopping, as by the above described plan.

*By Cauterization,*— This is a method which has been highly recommended, as a means of destroying the pulp, by some writers, (*Kœcker* and *Maury,*) the last cited author, observes, "We have pursued this plan for fifteen years with uniform success." I believe the reason why Mr. Maury met with so much success is, that in the use of the cautery, in any form, the vitality of the pulp is destroyed, and most generally removed more or less from the tooth, especially when the hot wire is used. Sometimes cold wire is thrust down the roots of the tooth so far as to be stopped by the diminished size of the canal; and in this way it is, also, removed far down in the root. The more there is removed of the pulp the better; for if only the minute extremity of the artery be left, it will contract and retract with more energy than if it were divided into numerous small branches; it is, also, distributed extensively over and through the pulp, and forms one of its principal constituents. When the pulp is exposed by removing the decay, and no matter how carefully, blood and lymph will ooze out immediately, and continue for an indefinite length of time, defying the permanent, effects of astringents; but if it be removed as far down in the roots as a small instrument can well be passed, the bleeding will cease in a few minutes, or hours at the farthest, and it is not of much importance in a pathological point of view, whether this be done by thrusting into the roots, a hot or cold instrument; the pulp is destroyed in either instance, and this is the principal indication to be met in the treatment. We can, and justly too, compare the exposed pulp to a small and extremely vascular tumor, the mere puncture of which, would establish an irrepressible hemorrhage ; but cut away the whole mass, and one single act of *torsion* upon the main trunk would immediately arrest it. From my experience, the *actual* cautery is the best means of destroying the pulp, where it can be properly applied, as in cases of the roots of the front teeth, where it needs but one or two applications to remove the whole pulp ; and I can affirm, with Mr. Maury, that I always succeed in my treatment of a tooth, where I can apply it properly ; but I seldom use it now, except in front roots, preparatory to setting teeth on pivots ; and the gum does not swell after the operation, as is common, when this method of supplying teeth is resorted to. Inflammation does not often follow the proper application of the actual cautery, if it is not too large, and applied too often ; but if it comes in contact with the walls, of the internal cavity, and be retained for an instant, it will exalt the temperature of the root so much as to inflame the alveola-dental membranes, (and of course, abscess *may* be the result,) it therefore requires great care in its application.—Some merely "*touch lightly*" the pulp, so as to produce an *eschar;* in so doing, the whole pulp in many cases becomes highly inflamed, and causes intense pain, because the eschar or shriveled spot acts to contract the remaining and living part of the pulp, in a similar manner, as if it were grasped with a small pincer, or were pressed upon by a plug ; besides the deadened part acts as a foreign body, and produces inflammation.

If in such cases, the entire pulp be removed, or destroyed, the pain ceases. Mr. Bell, of London, deprecates the use of the actual cautery, as well as all corrosive acids. He says: "The first and speedy effect of their application, is to produce extreme inflammation in the membrane, (*he means the dental pulp,*) with such intense suffering, as to demand the immediate removal of the tooth." He abandons the use of any agents, therefore, which have a tendency to destroy the vitality of the pulp, as improper ; and recommends a method of treatment by *stimulants*, which I would be pleased to continue in my next.

